# Validation of the Decipher Test for predicting adverse pathology in candidates for prostate cancer active surveillance

**DOI:** 10.1038/s41391-018-0101-6

**Published:** 2018-12-12

**Authors:** Hyung L. Kim, Ping Li, Huei-Chung Huang, Samineh Deheshi, Tara Marti, Beatrice Knudsen, Hatem Abou-Ouf, Ridwan Alam, Tamara L. Lotan, Lucia L. C. Lam, Marguerite du Plessis, Elai Davicioni, Neil Fleshner, Brian R. Lane, Ashley E. Ross, John W. Davis, James L. Mohler, Bruce J. Trock, Eric A. Klein, Jeffrey J. Tosoian, M. Eric Hyndman, Tarek A. Bismar

**Affiliations:** 1 Cedars-Sinai Medical Center, Los Angeles, CA USA; 2 GenomeDx Inc., Vancouver, BC Canada; 3 University of Calgary, Calgary, AB Canada; 4 Johns Hopkins Medical Institutions, Baltimore, MD USA; 5 University of Toronto, Toronto, ON Canada; 6 Spectrum Health, Grand Rapids, MI USA; 7 The University of Texas MD Anderson Cancer Center, Houston, TX USA; 8 Roswell Park, Buffalo, NY USA; 9 Cleveland Clinic Glickman Urology and Kidney Institute, Cleveland, OH USA

**Keywords:** Prognostic markers, Prostate cancer

## Abstract

**Background:**

Many men diagnosed with prostate cancer are active surveillance (AS) candidates. However, AS may be associated with increased risk of disease progression and metastasis due to delayed therapy. Genomic classifiers, e.g., Decipher, may allow better risk-stratify newly diagnosed prostate cancers for AS.

**Methods:**

Decipher was initially assessed in a prospective cohort of prostatectomies to explore the correlation with clinically meaningful biologic characteristics and then assessed in diagnostic biopsies from a retrospective multicenter cohort of 266 men with National Comprehensive Cancer Network (NCCN) very low/low and favorable-intermediate risk prostate cancer. Decipher and Cancer of the Prostate Risk Assessment (CAPRA) were compared as predictors of adverse pathology (AP) for which there is universal agreement that patients with long life-expectancy are not suitable candidates for AS (primary pattern 4 or 5, advanced local stage [pT3b or greater] or lymph node involvement).

**Results:**

Decipher from prostatectomies was significantly associated with adverse pathologic features (*p*-values < 0.001). Decipher from the 266 diagnostic biopsies (64.7% NCCN-very-low/low and 35.3% favorable-intermediate) was an independent predictor of AP (odds ratio 1.29 per 10% increase, 95% confidence interval [CI] 1.03–1.61, *p*-value 0.025) when adjusting for CAPRA. CAPRA area under curve (AUC) was 0.57, (95% CI 0.47–0.68). Adding Decipher to CAPRA increased the AUC to 0.65 (95% CI 0.58–0.70). NPV, which determines the degree of confidence in the absence of AP for patients, was 91% (95% CI 87–94%) and 96% (95% CI 90–99%) for Decipher thresholds of 0.45 and 0.2, respectively. Using a threshold of 0.2, Decipher was a significant predictor of AP when adjusting for CAPRA (*p*-value 0.016).

**Conclusion:**

Decipher can be applied to prostate biopsies from NCCN-very-low/low and favorable-intermediate risk patients to predict absence of adverse pathologic features. These patients are predicted to be good candidates for active surveillance.

## Introduction

Asymptomatic prostate cancers (PCa) can be managed without definitive local therapy [1, 2]. This is reflected in the National Comprehensive Cancer Network (NCCN) guidelines [3]. However, in randomized trials designed to assess the benefits of prostatectomy and radiotherapy [1, 2, 4], observation was associated with an increased risk of disease progression and metastasis. Furthermore, PCa remains the 2^nd^ most common cause of cancer-death in the United States [5]. Therefore, curative therapies remain an important option for patients at greatest risk for death from PCa, and better strategies to risk-stratify PCa are needed.

The majority of prostate cancers can be managed with active surveillance (AS), avoiding overtreatment to low-risk patients particularly in the era of early detection. Yet, most men in the US continue to pursue active therapy. In a large retrospective study utilizing the US national hospital based oncology database, Parikh et al. showed that only 14.2% of patients pursued AS in a cohort of 40,839 patients with very low-risk disease (defined as ≤ T1c; GS ≤ 6, PSA < 10; and positive biopsy cores < 33%) [6]. In this study, 85.8% underwent definitive local therapy. The underutilization of AS is due, in part, to concerns about misclassifying disease risk when using standard clinical parameters [7, 8]. This concern is highlighted by the observation that ~36% of low grade cancers based on biopsy have high grade disease following prostatectomy [9]. Patel et al. reported that 25% of patients with low volume GS 3 + 4 on biopsy harbor adverse pathology (AP), defined as GS at least 4+3, advanced local stage (pT3b or greater) or lymph node involvement (LNI), upon radical prostatectomy (RP) [7].

Molecular biomarkers are projected to play an increasing role in clinical decision-making for newly diagnosed, localized PCa [10–13]. Qualification studies provide additional validation and expand indications for previously reported biomarkers. Decipher® is a 22-feature RNA biomarker assay that was developed to predict metastasis following prostatectomy [14]. Decipher has been shown to predict metastasis and PCa-specific mortality from biopsy tissue [12, 13]. This study assesses a role for Decipher in predicting AP, which would make patients a poor candidate for AS.

## Materials & methods

### Study Cohort

#### Patient selection

First, a prospective cohort of 16,806 RP samples (referred to as RP Cohort) was used to illustrate the stratification of Decipher post-RP by pathological features. Prospectively collected patient samples from the Decipher PCa classifier test (GenomeDx Biosciences Laboratory, San Diego, CA) in the GRID™ were de-identified and aggregated for analysis. The GRID™ collects genomic expression data when the commercial Decipher test is ordered and provides the aggregated data for research use (NCT02609269). A waiver of informed consent was obtained from Western IRB (protocol #20172337).

Second, we identified 266 NCCN-very-low/low or favorable-intermediate risk PCa patients who underwent diagnostic prostate biopsy between 2000 and 2014 and were treated with RP in six community or academic practices (referred to as biopsy cohort): University of Calgary, Cedars-Sinai, Spectrum Health, Cleveland Clinic, MD Anderson Cancer Center, and Johns Hopkins. Sample size was maximized; no a priori sample size estimation was performed. Patients with complete tumor pathology from biopsy and prostatectomy and Decipher genomic expression profiles generated from diagnostic biopsy specimens were selected for analysis. Low-risk PCa was cT1c or cT2a, and Gleason score (GS) ≤ 6, and PSA < 10 ng/ml; favorable-Intermediate risk was no greater than predominant GS 3 and percent positive biopsy cores < 50%, and either cT2b-cT2c or PSA 10–20 ng/ml. Institutional review boards (IRB) at the participating institutions approved the research protocol.

#### Specimen collection and processing

Specimen selection and processing was performed by GenomeDx Biosciences Laboratory (San Diego, CA, USA) using their CLIA-certified commercial platform as described previously [12]. RNA was extracted from the needle biopsy core with the highest GS and percentage of tumor involvement. Priority was given to the cancer nodule with the highest GS. Following microarray quality control using the Affymetrix Power Tools packages [15], probeset summarization and normalization were performed using the single channel array normalization (SCAN) algorithm [16]. Microarray data was depositied in NCBI GEO Microarray repository (GSE119616).

Decipher, a 22-marker prognostic gene-expression score, was determined from the Decipher PCa classifier assay (GenomeDx Biosciences Laboratory, San Diego, CA, USA) as previously described [12, 14, 17]. Cancer of the Prostate Risk Assessment (CAPRA) scores were calculated as previously described [12, 18]. Risk-group categorizations of Decipher and CAPRA were based on prior publications [12, 18, 19].

### Statistical analysis

Box plots of Decipher and *p*-values resulted from Wilcoxon’s test in both cohorts were to demonstrate the association of Decipher with pathology features. Remaining analyses were based on the retrospective biopsy cohort. Descriptive statistics, medians, and ranges were reported for continuous variables and frequencies and proportions for categorical variables.

The primary objective of the study was to evaluate Decipher as an independent predictor of AP (defined as pT3b or greater, and/or primary Gleason pattern 4 or greater, and/or LNI) and to explore Decipher as a tool to identify a subgroup of patients likely to be free from AP. Univariable (UVA) and multivariable (MVA) logistic regression models were fitted with CAPRA and Decipher to evaluate Decipher as a prognostic indicator of AP compared with CAPRA. Firth’s penalization method was performed to account for the small number of events [20]. Odds ratio (OR), the corresponding 95% confidence interval and *p*-value were used for performance assessment. An area under the curve (AUC) [21] and bootstrapped 95% confidence interval were calculated through 1000 resamplings for each model. The incremental benefit of adding Decipher to a model with CAPRA was quantified by the difference in the AUCs. Optimism adjustment was performed on all MVAs to avoid overfitting in the models [22].

Generalized linear mixed models were used, treating institutions as the random intercepts [23] to account for potential confounding institutional effect. The primary analysis was repeated with time from biopsy to RP to adjust for biological progression over time. Decipher was compared to individual clinical risk factors and NCCN using univariable, multivariable logistic regression models, and AUCs.

Test characteristics of Decipher were summarized when using various cutoffs to dichotomize Decipher into a binary variable. Agreement metrics, specifically the negative predictive value (NPV), were used to investigate discriminatory ability for every Decipher score incremented by 0.05, which included the previously established Decipher low-risk cut-point of 0.45 [12, 13] and a lower-risk cut-point of 0.2 identified by Nguyen et al [13]. All NPVs were adjusted for 10% prevalence of AP [7] and the Wilson method was used to construct the 95% confidence intervals [24]. Logistic regression result of the 0.2 cut-point for predicting AP was performed as an example to demonstrate the discrimination provided by the genomic classifier.

All statistical tests were two-sided. *P*-values less than 0.05 were considered statistically significant. Analyses were performed in R, version 3.3.1 (R Foundation for Statistical Computing, Vienna, Austria).

## Results

### Association of Decipher score and AP for RP cohort

The distribution of Decipher was assessed from prospectively collected RPs to determine if Decipher is able to discriminate based on stage or grade (Supp. Figure 1). Decipher distributions were different when stratified by stage ( ≤ pT3a vs ≥ pT3b), primary Gleason grade ( ≤ 3 vs ≥ 4), LN status (negative vs positive) or the presence of any of these adverse pathologic features (*p*-values < 0.001). The median Decipher of patients with AP (*n* = 9356) and without AP (*n* = 6694) at RP were 0.62 and 0.45, respectively (*p*-value < 0.001).

### Patient characteristics for biopsy cohort

Decipher in the biopsy cohort were examined in 266 men with favorable NCCN risk (64.7% with very low/low-risk disease and 35.3% with favorable-intermediate risk, Table 1). The goal was to determine if biopsy Decipher can predict prostatectomy pathology in men with very low/low and favorable-intermediate PCa who are candidates for AS. The median age was 62 years and the median PSA at diagnosis was 5.4 ng/mL (interquartile range [IQR] 4.16ng/mL–7.19 ng/mL). The majority of patients (84.6%) were diagnosed with cT1 PCa and 75.6% patients were in biopsy grade group 1. 186 (69.9%) and 76 (28.6%) of the patients were classified as CAPRA low and intermediate risk, respectively. At prostatecomty, 32 (12%) had AP (pT3b/N1 or primary Gleason pattern 4 or higher). The rate of AP was 11% (19/172) and 14% (13/94) for the NCCN-very-low/low and favorable-intermediate patients, respectively. The median time from biopsy to RP was 2.2 months (IQR of 1.35 and 3.63). Twenty eight (10.5%) patients had grade group 3–5; 27 (10.2%) harbored primary Gleason pattern 4 or higher. Seventy one (26.7%) were pT3a and 5 (1.9%) were pT3b. Positive LNs were found in three patients (1.1%). Median Decipher in this population was 0.28 (IQR 0.17–0.39) and was significantly higher among men with AP (0.34 IQR 0.25–0.47 vs 0.27 IQR 0.15–0.37, *p*-value < 0.001, Supp. Figure 2).

**Table 1:** Clinical characteristics of active surveillance candidates in biopsy cohort

Variables	Summary statistics
No. patients	266
Age, year	
Median (Q1, Q3)	62 (58–67)
Race, n (%)	
African American	10 (3.8)
Caucasian	65 (24.4)
Other	43 (16.2)
Unavailable	148 (55.6)
Biopsy stage, n (%)	
cT1	225 (84.6)
cT2	41 (15.4)
Biopsy Grade Group, n (%)	
Grade 1	201 (75.6)
Grade 2	65 (24.4)
% positive biopsy cores	
Median (Q1, Q3)	30 (23.7, 40; NA = 2)
PSA at enrollment, ng/mL	
Median (Q1, Q3)	5.4 (4.16, 7.19)
NCCN, n (%)	
Low	172 (64.7)
Favorable Intermediate	94 (35.3)
Categorical CAPRA, n (%)	
Low	186 (69.9)
Int	76 (28.6)
Unavailable	4 (1.5)
Time from biopsy to RP, month	
Median (Q1, Q3)	2.2 (1.35, 3.63)
RP stage, n (%)	
pT2c or less	190 (71.4)
pT3a	71 (26.7)
pT3b	5 (1.9)
RP Grade Group, n (%)	
Grade 1	94 (35.3)
Grade 2	144 (54.1)
Grade 3	23 (8.6)
Grade 4	4 (1.5)
Grade 5	1 (0.4)
Positive surgical margins, n (%)	
Present	58 (21.8)
Absent	208 (78.2)
Lymph node invasion, n (%)	
Present	3 (1.1)
Absent	263 (98.9)
Follow-up for censored patients, year	
Median (Q1, Q3)	5.32 (2.36, 9.22)

*Q1* first quartile, *Q3* third quartile, *NA* not available, *RP* radical prostatectomy

### Performance of Decipher and CAPRA for predicting AP in biopsy cohort

Decipher was an independent predictor of AP (Table 2). In UVA, Decipher was a predictor of AP with an OR of 1.32 (95% CI 1.07–1.63, *p*-value 0.011). In MVA when adjusting for CAPRA, Decipher was an independent predictor with an OR of 1.29 (95% CI 1.03–1.61, *p*-value 0.025). CAPRA was not a significant predictor of AP in either UVA (*p*-value 0.109) or MVA (*p*-value 0.239). When used alone, CAPRA had an AUC of 0.57 (95% CI 0.47–0.68). The MVA model of CAPRA and Decipher had an AUC of 0.65 (95% CI 0.58–0.70) after adjusting for optimism. Adding Decipher improved the AUC by 0.08 (Table 2). Receiver Operating Characteristics (ROC) curves of the models are shown in Supp. Figure 3.

**Table 2:** Logistic regression analysis for predicting adverse pathology in biopsy cohort

Model	Variable	Odds ratio (95% CI)	*P*-value	AUC (95% CI)
Univariable	CAPRA	1.42 (0.93–2.19)	0.109	0.57 (0.47–0.68)
	Decipher	1.32 (1.07–1.63)	0.011*	0.65 (0.56–0.74)
Multivariable: CAPRA + Decipher	CAPRA	1.29 (0.84–2.00)	0.239	0.65 (0.58–0.70)^a^
	Decipher	1.29 (1.03–1.61)	0.025*	

Odds ratios of Decipher were reported per 0.1 unit increase. 4 patients without CAPRA scores were excluded from models with CAPRA.

*P-value < 0.05

^a^AUC was adjusted for optimism

*CI* confidence interval, *AUC* area under curve

Similar results were found when the performance of Decipher for predicting AP accounted for institution (MVA OR 1.29, 95% CI 1.02–1.64, *p*-value 0.034, Supp. Table 1) and time from biopsy to RP (MVA OR 1.29, 95% CI 1.03–1.62, *p*-value 0.027, Supp. Table 2); similar effect sizes indicate the robustness of our models. Moreover, Decipher increased the AUC of NCCN from 0.53 to 0.64 when added to the NCCN model (Supp. Table 3; ROC curves in Supp. Figure 4). Additional UVA and MVA results comparing Decipher with individual clinical risk factors in CAPRA and NCCN can be found in Supp. Table 3; only Decipher was a statistically significant predictor of AP in both UVA and MVA.

### Decipher for predicting AP in biopsy cohort

The sensitivities and specificities of various Decipher thresholds were evaluated to predict AP in radical prostatcomy (Table 3). For example, 17.7% of patients had Decipher > 0.45 and 19.1% had AP in this group, compared to 10.5% with AP for patients with Decipher ≤ 0.45. The sensitivity and specificity for predicting AP with this cutoff were 28% and 84%, respectively. At a threshold of 0.2, 66.9% of patients were in the high-risk group for AP, with a sensitivity of 88% and specificity of 36%. For patients with Decipher > 0.2, the AP rate was 15.7%, but for patients with a score ≤ 0.2, the AP rate improved to 4.5%.

**Table 3:** Sensitivity and specificity of Decipher risk thresholds for predicting AP in biopsy cohort

Cut point	Proportion (%)^a^	Sensitivity (95% CI)	Specificity (95% CI)
0.45	17.7	28% (16–45%)	84% (78–88%)
0.40	22.9	34% (20–52%)	79% (73–83%)
0.35	32.3	50% (34–66%)	70% (64–76%)
0.30	45.5	56% (39–72%)	56% (50–62%)
0.25	57.5	78% (61–89%)	45% (39–52%)
0.20	66.9	88% (72–95%)	36% (30–42%)

^a^Proportion of patients with Decipher score greater than the cut point

*CI* confidence interval

**Fig. 1:**
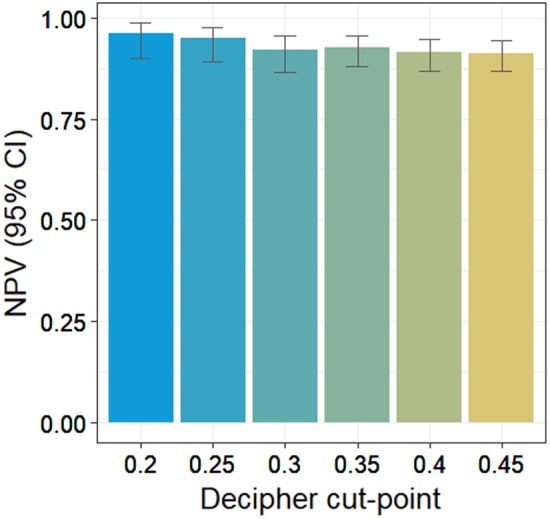
Negative predictive value (NPV) for the absence of AP at varying Decipher risk thresholds in biopsy cohort

When considering AS, the NPV is useful as it determines the degree of confidence no AP at RP for a specific patient. Fig. 1 provides a histogram of NPVs as a function of various Decipher thresholds; for thresholds of 0.45 and 0.2, the NPV were 91% and 96%, respectively. Given the high NPV associated with a threshold of 0.2, a Decipher ≤ 0.2 would provide a strong case for patients considering AS. A Decipher risk group defined by a cut-point of 0.2 showed its prognostic potential (Table 4), statistically significant in both UVA (*p*-value 0.006) and MVA (*p*-value 0.016) for predicting AP. Patients with Decipher greater than 0.2 were more likely to have AP (OR 3.17, 95% CI 1.22–10.26) than patients with Decipher ≤ 0.2.

**Table 4:** Logistic regression analysis for predicting adverse pathology using Decipher cut-point of 0.2 in biopsy cohort

Model	Variable	Odds ratio (95% CI)	*P*-value
Univariable	CAPRA	1.42 (0.93–2.19)	0.109
	Categorical CAPRA Int. vs Low	1.51 (0.67–3.26)	0.311
	Decipher	1.32 (1.07–1.63)	0.011*
	Decipher > 0.2 vs ≤ 0.2	3.56 (1.39–11.43)	0.006*
Multivariable: CAPRA + Decipher	CAPRA	1.29 (0.84–2.00)	0.239
	Decipher	1.29 (1.03–1.61)	0.025*
Multivariable: Categorical CAPRA + Categorical Decipher (cutpoint = 0.2)	Categorical CAPRA Int. vs Low	1.31 (0.58–2.86)	0.513
	Decipher > 0.2 vs ≤ 0.2	3.17 (1.22–10.26)	0.016*

Odds ratios of Decipher were reported per 0.1 unit increase. 4 patients without CAPRA scores were excluded in models with CAPRA.

*P-value < 0.05

*CI* confidence interval

## Discussion

Molecular classification tools are helpful if they assist with patient decision-making by providing information beyond what is available from clinical variables. Although AS is now a well-accepted option for the majority of newly diagnosed low grade prostate cancers, the risk of understaging and undergrading prostate cancer remains a concern. The NCCN guidelines recommend that AS be considered for even favorable-intermediate risk patients. Therefore, we focused on the risk of pathologic findings at prostatectomy that would place a patient in a higher risk group. We used a previously characterized definition for AP that is universally accepted as being inappropriate for AS: pT3b or higher, GS at least 4 + 3 = 7 or LN metastasis [7]. The objective of this study was to determine if Decipher, which is commercially available and developed for predicting clinical metastasis following prostatectomy, can be used to stratify the risk for AP using diagnostic biopsy tissue.

When the Decipher test was applied to prostatectomy tissue, there was a strong association between the genomic score and each of the individual pathologic features defining AP. Therefore, the 22-transcript Decipher test reflects the biology that produces AP. However, the question remains whether Decipher can be applied to the diagnostic biopsy tissue to predict AP. Therefore, we identified a retrospective cohort of men who underwent RP based on historic treatment standards, but who are considered AS candidates by contemporary standards. In this group, Decipher from the prostate biopsy was a significant predictor of AP when used alone, or in MVA analysis with CAPRA, NCCN, or the individual elements of these clinical risk-stratification tools. In contrast, none of the clinical variables or risk-stratification tools were significant predictors of AP, but this is not surprising since our cohort is homogenous, including only AS candidates at low risk for harboring aggressive PCa. Accordingly, we observed the overall 12% AP event rate was not significantly different between NCCN very low/low risk (11%) and favorable-intermediate risk (14%) subgroups. We did, however, show that the AUC for Decipher was better than both CAPRA and NCCN. For such a homogenous cohort, an AUC of 0.65 for Decipher can be considered meaningful in this setting.

For newly diagnosed patients, a molecular test that can increase confidence in the absence of AP can increase acceptance of AS. To support this binary clinical decision, Decipher can be converted to a binary variable by applying a threshold. Of all patients in our biopsy cohort, 88% were free of AP. A useful molecular test should be able to identify a low-risk group where the likelihood of being free of AP is greater than 88%. In other words, the NPV should be above 88%. Thresholds of 0.45 and 0.2 have been reported and therefore their use avoids the risk of overfitting associated with exploring new cutoffs. A cutoff of ≤ 0.45 had a NPV of 91% and included 82.3% of the cohort. A cutoff of ≤ 0.2 had a NPV of 96% but included 33.1% of the cohort. A practical way to apply these cutoffs is to strongly recommend AS for scores ≤ 0.2 since the risk of AP is only 5%, and recommend definitive local therapy for scores > 0.45 since the risk of AP is 19%. Therefore, the Decipher test would result in a strong recommendation for or against AS in ~50% of patients considering AS solely based on clinical parameters.

Our study has several important strengths. It qualifies Decipher for prediction of AP from the prostate biopsy specimen. It uses a well-characterized commercial assay readily available for clinical use, and cutoffs for clinical decision-making that have been reported. It is a multi-institutional study that reduces the risk of bias that can result from institution-specific protocols for specimen handling and storage. We take advantage of tissue collected from an era where it was acceptable to recommend prostatectomy to all men with PCa and a cohort of modern AS candidates where both biopsy tissue and prostatectomy pathology are available. In the modern era, a prospective study of this type would not be ethical. Finally, rather than predict any degree of upgrading or upstaging, we predict a very high-risk group where there is universal acceptance that AS is inappropriate. This study did not have long-term follow-up to consider survival outcomes and the sample size and low number of events did not allow Decipher to be assessed in individual NCCN risk (e.g., favorable intermediate only) risk groups. An ongoing multi-institutional study of favorable-intermediate risk patients aims to address this limitation.

## Conclusion

Decipher can be applied to prostate biopsies from NCCN-very-low/low and favorable-intermediate risk patients to predict AP found in prostatectomy pathology that would make a patient an inappropriate candidate for AS. Decipher’s high NPV for identifying patients without AP can provide reassurance for AS.

## Electronic supplementary material

Supplementary Tables and Figures Legends

Supp. Table 1

Supp. Table 2

Supp. Table 3

Supp. Fig. 1A

Supp. Fig. 1B

Supp. Fig. 1C

Supp. Fig. 1D

Supp. Fig. 2

Supp. Fig. 3

Supp. Fig. 4A

Supp. Fig. 4B

Supp. Fig. 4C

## References

[1] Hamdy FC, Donovan JL, Lane JA, Mason M, Metcalfe C, Holding P (2016). 10-year outcomes after monitoring, surgery, or radiotherapy for localized prostate cancer. N Engl J Med.

[2] Wilt TJ, Jones KM, Barry MJ, Andriole GL, Culkin D, Wheeler T (2017). Follow-up of prostatectomy versus observation for early prostate cancer. N Engl J Med.

[3] Lee RJ, Dana-Farber V-C, Antonarakis ES, Armstrong AJ, Victor AD, Davis BJ, et al. Prostate Health Education Network (PHEN) NCCN Guidelines Version 3. p30 2018 Prostate Cancer.

[4] Bill-Axelson A, Holmberg L, Garmo H, Rider JR, Taari K, Busch C (2014). Radical prostatectomy or watchful waiting in early prostate cancer. N Engl J Med.

[5] Siegel RL, Miller KD, Jemal A (2018). Cancer statistics, 2018. CA Cancer J Clin.

[6] Parikh RR, Kim S, Stein MN, Haffty BG, Kim IY, Goyal S (2017). Trends in active surveillance for very low-risk prostate cancer: do guidelines influence modern practice?. Cancer Med.

[7] Patel HD, Tosoian JJ, Carter HB, Epstein JI (2018). Adverse pathologic findings for men electing immediate radical prostatectomy. JAMA Oncol.

[8] Gearman DJ, Morlacco A, Cheville JC, Rangel LJ, Karnes RJ (2018). Comparison of pathological and oncologic outcomes of favorable risk gleason score 3 + 4 and low risk gleason score 6 prostate cancer: considerations for active surveillance. J Urol.

[9] Epstein JI, Feng Z, Trock BJ, Pierorazio PM (2012). Upgrading and downgrading of prostate cancer from biopsy to radical prostatectomy: incidence and predictive factors using the modified Gleason grading system and factoring in tertiary grades. Eur Urol.

[10] Cuzick J, Swanson GP, Fisher G, Brothman AR, Berney DM, Reid JE (2011). Prognostic value of an RNA expression signature derived from cell cycle proliferation genes in patients with prostate cancer: a retrospective study. Lancet Oncol.

[11] Cullen J, Rosner IL, Brand TC, Zhang N, Tsiatis AC, Moncur J (2015). A biopsy-based 17-gene genomic prostate score predicts recurrence after radical prostatectomy and adverse surgical pathology in a racially diverse population of men with clinically low- and intermediate-risk prostate cancer. Eur Urol.

[12] Klein EA, Haddad Z, Yousefi K, Lam LLC, Wang Q, Choeurng V (2016). Decipher genomic classifier measured on prostate biopsy predicts metastasis risk. Urol.

[13] Nguyen PL, Martin NE, Choeurng V, Palmer-Aronsten B, Kolisnik T, Beard CJ (2017). Utilization of biopsy-based genomic classifier to predict distant metastasis after definitive radiation and short-course ADT for intermediate and high-risk prostate cancer. Prostate Cancer Prostatic Dis.

[14] Erho N, Crisan A, Vergara IA, Mitra AP, Ghadessi M, Buerki C (2013). Discovery and validation of a prostate cancer genomic classifier that predicts early metastasis following radical prostatectomy. PLoS ONE.

[15] Lockstone HE (2011). Exon array data analysis using Affymetrix power tools and R statistical software. Brief Bioinform.

[16] Piccolo SR, Sun Y, Campbell JD, Lenburg ME, Bild AH, Johnson WE (2012). A single-sample microarray normalization method to facilitate personalized-medicine workflows. Genomics.

[17] Karnes RJ, Bergstralh EJ, Davicioni E, Ghadessi M, Buerki C, Mitra AP (2013). Validation of a genomic classifier that predicts metastasis following radical prostatectomy in an at risk patient population. J Urol.

[18] Cooperberg MR, Broering JM, Carroll PR (2009). Risk assessment for prostate cancer metastasis and mortality at the time of diagnosis. J Natl Cancer Inst.

[19] Ross AE, Johnson MH, Yousefi K, Davicioni E, Netto GJ, Marchionni L (2016). Tissue-based genomics augments post-prostatectomy risk stratification in a natural history cohort of intermediate- and high-risk men. Eur Urol.

[20] Firth D (1993). Bias reduction of maximum likelihood estimates. Biometrika.

[21] Bradley AP (1997). The use of the area under the ROC curve in the evaluation of machine learning algorithms. Pattern Recognit.

[22] Smith GCS, Seaman SR, Wood AM, Royston P, White IR (2014). Correcting for optimistic prediction in small data sets. Am J Epidemiol.

[23] Hedeker D Generalized Linear Mixed Models. In: BS Everitt and DC Howell editors. Encyclopedia of Statistics in Behavioral Science. New Jersey: John Wiley & Sons; 2005.

[24] DasGupta A Normal Approximations and the Central Limit Theorem. In: Fundamentals of Probability: A First Course. p. 213–42. Springer, New York, NY: Springer Texts in Statistics; 2010.

