# Supplementary material for: Validation of the Decipher Test for predicting adverse pathology in candidates for prostate cancer active surveillance
Source: Prostate Cancer Prostatic Dis. 2018 Dec 12;22(3):399–405. doi: 10.1038/s41391-018-0101-6 (PMC6760567; doi:10.1038/s41391-018-0101-6)
Supplement: Supplementary file 1 — Supplementary Tables and Figures Legends [file 41391_2018_101_MOESM1_ESM.docx]

**SUPPLEMENTARY TABLES**

Supp. Table 1 Logistic regression analysis for predicting adverse pathology in Biopsy Cohort, adjusting for institution

Supp. Table 2 Logistic regression analysis for predicting adverse pathology in Biopsy Cohort, adjusting for time from biopsy to RP

Supp. Table 3 Logistic regression analysis for predicting adverse pathology in Biopsy Cohort, adjusting for individual clinical risk factors or NCCN

**SUPPLEMENTARY FIGURES**

Supp. Figure 1 Association of Decipher and adverse pathology features in RP cohort A) pT category; B) primary Gleason pattern; C) lymph node status; D) adverse pathology

Supp. Figure 2 Association of Decipher and adverse pathology features in Biopsy Cohort

Supp. Figure 3 AUCs in Biopsy Cohort for adverse pathology of CAPRA and Decipher († indicates optimism adjusted AUC)

Supp. Figure 4 AUCs in Biopsy Cohort for adverse pathology of A) Individual clinical risk factors (UVA); B) Individual clinical risk factors and Decipher; C) NCCN and Decipher († indicates optimism adjusted AUC)
