# Supplementary material for: Validation of the Decipher Test for predicting adverse pathology in candidates for prostate cancer active surveillance
Source: Prostate Cancer Prostatic Dis. 2018 Dec 12;22(3):399–405. doi: 10.1038/s41391-018-0101-6 (PMC6760567; doi:10.1038/s41391-018-0101-6)
Supplement: Supplementary file 2 — Supp. Table 1 [file 41391_2018_101_MOESM2_ESM.docx]

| **Model** | **Variable** | **Odds ratio (95% CI)** | **P-value** |
| --- | --- | --- | --- |
| Univariable | CAPRA | 1.45 (0.93 - 2.25) | 0.104 |
|  | Decipher | 1.32 (1.05 - 1.67) | 0.018* |
| Multivariable: CAPRA + Decipher | CAPRA | 1.33 (0.85 - 2.08) | 0.218 |
|  | Decipher | 1.29 (1.02 - 1.64) | 0.034* |
| *Odds ratios of Decipher were reported per 0.1 unit increased.* | | | |
| *4 patients were excluded in models when CAPRA was considered.* | | | |
| *Institutions were adjusted in generalized linear mixed models as the random intercepts.* | | | |
| *Institutions with less than 10 samples were removed in the analysis.* | | | |
| ** P-value < 0.05.* |  |  |  |
| *Abbreviations: CI = confidence interval.* | | | |
