# Supplementary material for: Validation of the Decipher Test for predicting adverse pathology in candidates for prostate cancer active surveillance
Source: Prostate Cancer Prostatic Dis. 2018 Dec 12;22(3):399–405. doi: 10.1038/s41391-018-0101-6 (PMC6760567; doi:10.1038/s41391-018-0101-6)
Supplement: Supplementary file 3 — Supp. Table 2 [file 41391_2018_101_MOESM3_ESM.docx]

| **Model** | **Variable** | **Odds ratio (95% CI)** | **P-value** |
| --- | --- | --- | --- |
| Univariable | Time from biopsy to RP | 1.14 (1.04 - 1.26) | 0.007* |
| Multivariable: CAPRA + Time from biopsy to RP | CAPRA | 1.31 (0.85 - 2.04) | 0.221 |
|  | Time from biopsy to RP | 1.12 (1.01 - 1.24) | 0.026* |
| Multivariable: Decipher + Time from biopsy to RP | Decipher | 1.31 (1.06 - 1.63) | 0.014* |
|  | Time from biopsy to RP | 1.14 (1.03 - 1.25) | 0.009* |
| Multivariable: CAPRA + Decipher + Time from biopsy to RP | CAPRA | 1.19 (0.77 - 1.86) | 0.429 |
|  | Decipher | 1.29 (1.03 - 1.62) | 0.027* |
|  | Time from biopsy to RP | 1.12 (1.01 - 1.24) | 0.029* |
| *Odds ratios of Decipher were reported per 0.1 unit increased; odds ratios of Time from biopsy to RP were reported per 1 month increased.* | | | |
| *4 patients were excluded in models when CAPRA was considered.* | | | |
| ** P-value < 0.05.* |  |  |  |
| *Abbreviations: CI = confidence interval.* | | | |
