# Supplementary material for: Validation of the Decipher Test for predicting adverse pathology in candidates for prostate cancer active surveillance
Source: Prostate Cancer Prostatic Dis. 2018 Dec 12;22(3):399–405. doi: 10.1038/s41391-018-0101-6 (PMC6760567; doi:10.1038/s41391-018-0101-6)
Supplement: Supplementary file 4 — Supp. Table 3 [file 41391_2018_101_MOESM4_ESM.docx]

| **Model** | **Variable** | **Odds ratio (95% CI)** | **P-value** | **AUC (95% CI)** |
| --- | --- | --- | --- | --- |
| Univariable | Age | 1.01 (0.96 - 1.07) | 0.647 | 0.54 (0.43-0.65) |
|  | log2 PSA | 1.65 (0.96 - 2.85) | 0.077 | 0.61 (0.51-0.72) |
|  | Biopsy Grade Group 2 vs. 1 | 0.89 (0.35 - 2.02) | 0.785 | 0.51 (0.44-0.59) |
|  | Biopsy stage cT2a-c vs. cT1 | 1.71 (0.66 - 4.01) | 0.255 | 0.54 (0.46-0.61) |
|  | % positive biopsy cores 1/3 or more vs. <1/3 | 1.41 (0.67 - 3.13) | 0.371 | 0.54 (0.45-0.63) |
|  | NCCN Fav. Int. vs. Low | 1.30 (0.61 - 2.73) | 0.488 | 0.53 (0.44-0.62) |
|  | Decipher | 1.32 (1.07 - 1.63) | 0.011* | 0.65 (0.56-0.74) |
| Multivariable: Age + Biopsy stage + log2 PSA + Biopsy Grade Group + % positive biopsy cores | Age | 0.99 (0.93 - 1.05) | 0.68 | 0.55 (0.49-0.56) † |
|  | Biopsy stage cT2a-c vs. cT1 | 1.80 (0.68 - 4.34) | 0.221 |  |
|  | log2 PSA | 1.65 (0.93 - 2.98) | 0.105 |  |
|  | Biopsy Grade Group 2 vs. 1 | 1.02 (0.39 - 2.42) | 0.959 |  |
|  | % positive biopsy cores 1/3 or more vs. <1/3 | 1.35 (0.63 - 3.01) | 0.442 |  |
| Multivariable: Age + Biopsy stage + log2 PSA + Biopsy Grade Group + % positive biopsy cores + Decipher | Age | 0.99 (0.93 - 1.06) | 0.776 | 0.62 (0.57-0.65) † |
|  | Biopsy stage cT2a-c vs. cT1 | 1.65 (0.61 - 4.05) | 0.306 |  |
|  | log2 PSA | 1.44 (0.89 - 2.61) | 0.293 |  |
|  | Biopsy Grade Group 2 vs. 1 | 1.00 (0.37 - 2.39) | 0.997 |  |
|  | % positive biopsy cores 1/3 or more vs. <1/3 | 1.48 (0.69 - 3.36) | 0.319 |  |
|  | Decipher | 1.27 (1.02 - 1.59) | 0.032* |  |
| Multivariable: NCCN + Decipher | NCCN Fav. Int. vs. Low | 1.21 (0.56 - 2.55) | 0.623 | 0.64 (0.57-0.68) † |
|  | Decipher | 1.31 (1.06 - 1.62) | 0.013* |  |
| *Odds ratios of Decipher were reported per 0.1 unit increased.* | | | | |
| *2 patients were excluded in models when % positive biopsy cores was considered.* | | | | |
| *† AUC was adjusted for optimism.* | | | | |
| ** P-value < 0.05.* |  |  |  |  |
| *Abbreviations: CI = confidence interval; AUC = area under curve; Fav. Int. = Favorable Intermediate.* | | | | |
