# Supplementary figures and images for: Validation of the Decipher Test for predicting adverse pathology in candidates for prostate cancer active surveillance
Source: Prostate Cancer Prostatic Dis. 2018 Dec 12;22(3):399–405. doi: 10.1038/s41391-018-0101-6 (PMC6760567; doi:10.1038/s41391-018-0101-6)

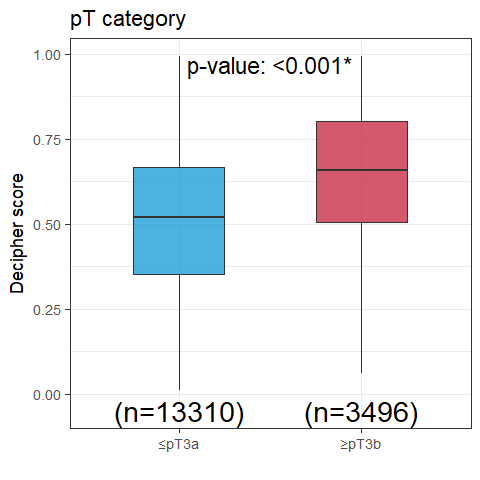

Supplement: Supplementary file 5 — Supp. Fig. 1A [file 41391_2018_101_MOESM5_ESM.tif]

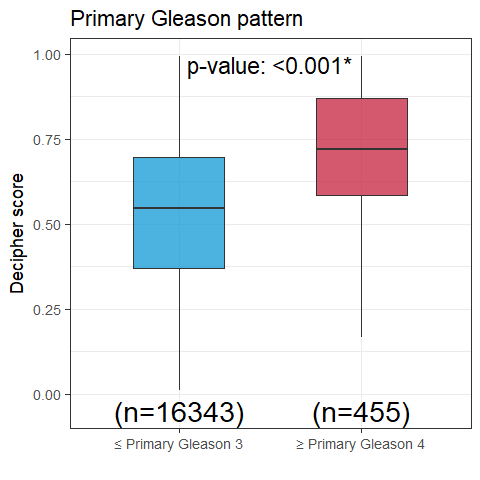

Supplement: Supplementary file 6 — Supp. Fig. 1B [file 41391_2018_101_MOESM6_ESM.tif]

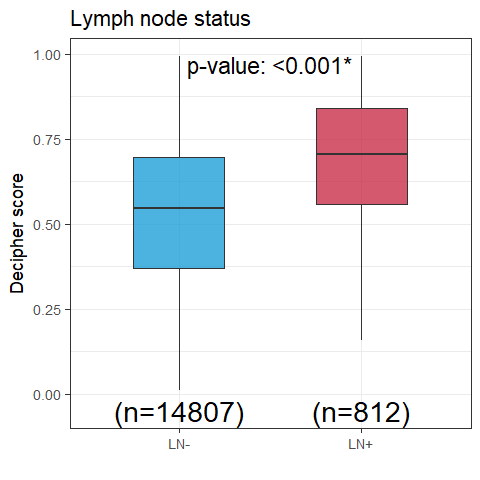

Supplement: Supplementary file 7 — Supp. Fig. 1C [file 41391_2018_101_MOESM7_ESM.tif]

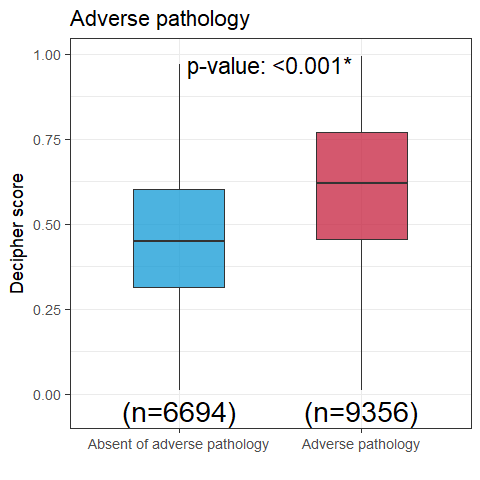

Supplement: Supplementary file 8 — Supp. Fig. 1D [file 41391_2018_101_MOESM8_ESM.tif]

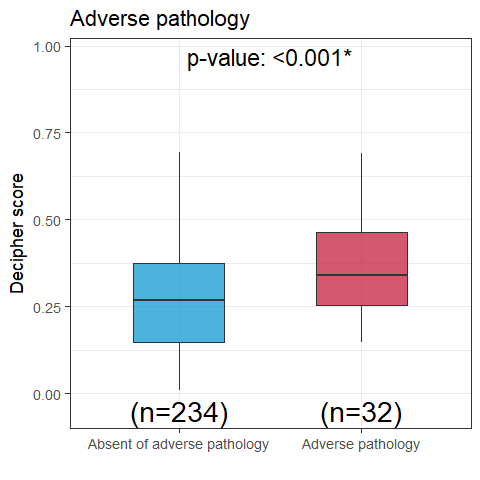

Supplement: Supplementary file 9 — Supp. Fig. 2 [file 41391_2018_101_MOESM9_ESM.tif]

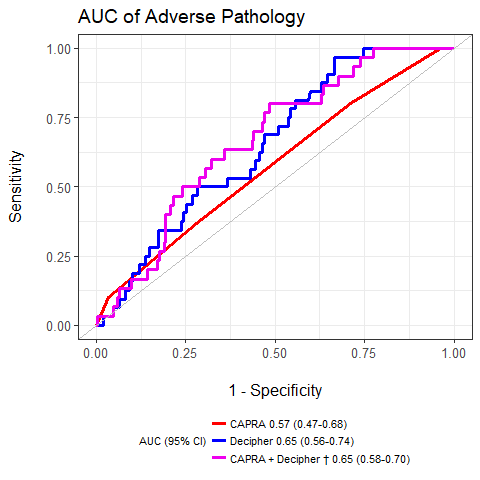

Supplement: Supplementary file 10 — Supp. Fig. 3 [file 41391_2018_101_MOESM10_ESM.tif]

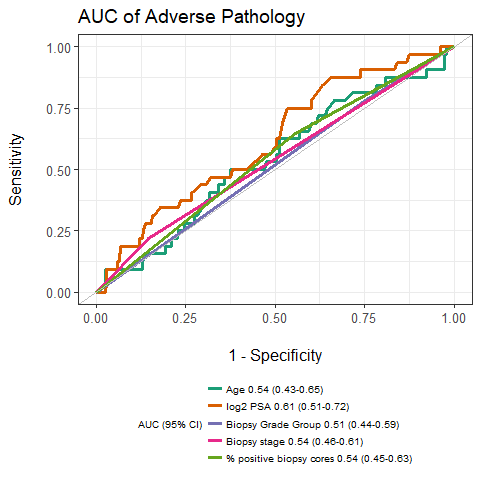

Supplement: Supplementary file 11 — Supp. Fig. 4A [file 41391_2018_101_MOESM11_ESM.tif]

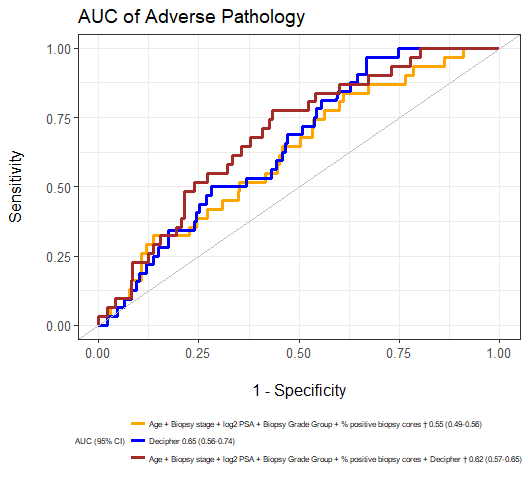

Supplement: Supplementary file 12 — Supp. Fig. 4B [file 41391_2018_101_MOESM12_ESM.tif]

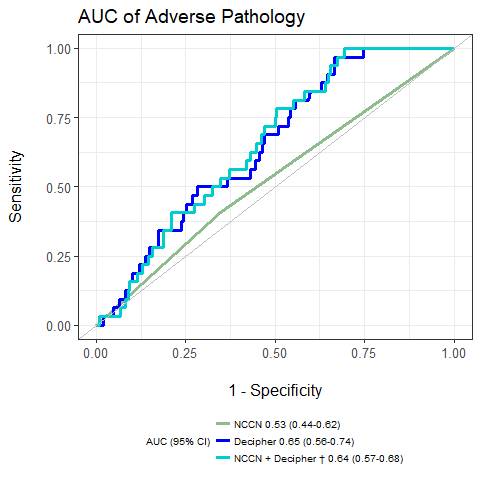

Supplement: Supplementary file 13 — Supp. Fig. 4C [file 41391_2018_101_MOESM13_ESM.tif]
